# Iodine Concentration in Brazilian Drinking Water and Its Possible Contribution to Iodine Intake for Different Physiological Groups

**DOI:** 10.1155/2022/7542632

**Published:** 2022-12-21

**Authors:** Carina Aparecida Pinto, Edimar Aparecida Filomeno Fontes, Sandra Patricia Crispim, Sarah Aparecida Vieira Ribeiro, Sylvia do Carmo Castro Franceschini, Nathalia Pizato, Franciane Rocha de Faria, Renata Junqueira Pereira, Carolina Abreu de Carvalho, Míriam Carmo Rodrigues Barbosa, Naiara Sperandio, Mariana de Souza Macedo, Silvia Eloiza Priore

**Affiliations:** ^1^Department of Nutrition and Health, Universidade Federal de Viçosa (UFV), Viçosa 36570900, Brazil; ^2^Department of Food Technology, Universidade Federal de Viçosa (UFV), Viçosa 36570900, Brazil; ^3^Department of Nutrition, Universidade Federal do Paraná (UFPR), Curitiba 80210170, Brazil; ^4^Department of Nutrition, Universidade Brasília (UnB), Brasília 70910900, Brazil; ^5^Department of Medicine, Universidade Federal de Rondonópolis (UFR), Rondonópolis 78735910, Brazil; ^6^Department of Health Sciences, Universidade Federal do Tocantins (UFT), Palmas 77001090, Brazil; ^7^Department of Medicine, Universidade Federal do Maranhão (UFMA), São Luís 65200000, Brazil; ^8^Department of Integrated Health Education, Universidade Federal do Espírito Santo (UFES), Vitória 29040090, Brazil; ^9^Institute of Food and Nutrition, Universidade Federal do Rio de Janeiro (UFRJ), Macaé 27930560, Brazil; ^10^Graduate Program in Nutrition Science, Universidade Federal dos Vales do Jequitinhonha e Mucuri (UFVJM), Diamantina 39100000, Brazil

## Abstract

**Objective:**

The objective is to analyze the concentration of iodine in Brazilian drinking water and its possible contribution to iodine intake for different groups.

**Methods:**

Water samples collected from primary healthcare units in eight locations distributed across all five macroregions of Brazil were analyzed. The quantification of iodine in the water samples was done by spectrophotometry (leuco crystal violet method). To classify the degree of iodine concentration, the recommendation of the Ministry of Health (China) was followed since Brazil lacks a classification standard. To verify the possible contribution of drinking water to iodine intake for different groups, the recommended water intake for each group according to the United States Institute of Medicine (2004) was considered. The percentage of iodine in drinking water and its contribution to iodine intake for different physiological groups were calculated based on the estimated average requirement (EAR) of iodine. A descriptive statistical analysis was performed using SPSS version 21.0 and Statistical Analysis Systems (SAS) version 9.2.

**Results:**

Significant differences were found between the maximum and minimum concentrations of iodine in water samples from the same location. In Pinhais (south region), the difference was 44.32 *μ*g· L^−1^; in Viçosa (southeast region), it was 27.86 *μ*g·L^−1^; in Rondonópolis (midwest region), it was 12.66 *μ*g·L^−1^; in São Luís (northeast region), it was 11.82 *μ*g·L^−1^; in Brasilian Federal District (midwest region), it was 10.98 *μ*g·L^−1^; in Macaé (southeast region), it was 10.14 *μ*g· L^−1^; in Palmas (north region), it was 4.22 *μ*g·L^−1^; and in Vitória (southeast region), it was 1.69 *μ*g·L^−1^. The maximum concentrations of iodine found in the drinking water of Pinhais and Viçosa can contribute more than 70.0% and 50.0%, respectively, to daily iodine intake for all groups.

**Conclusion:**

Monitoring the concentration of iodine in drinking water from different locations in each city or Federal District is a preventive measure against inadequate iodine intake and possible adverse changes in population health.

## 1. Introduction

Iodine is an essential micronutrient and a principal component of thyroid hormones. Inadequate, insufficient, or excessive iodine intake can modify the thyroid gland function [1, 2].

Iodine deficiency causes goiter, irreversible brain damage in fetus, known as cretinism, and developmental and physical growth delays in children. Among pregnant women, the main outcomes are abortion, stillbirth, congenital anomalies, and increased perinatal mortality [3]. On the other hand, excessive iodine intake can result in thyroid diseases, such as nodular goiter, hyperthyroidism, and Hashimoto's thyroiditis [1, 4].

Cases of goiter due to excess iodine in drinking water were initially reported by China [5]. Excess iodine in drinking water, especially concentrations of 300 to 1.300 *μ*g·L^−1^, has been associated with excessive iodine intake and high prevalence of endemic goiter in individuals aged 8 to 10 years [6–8].

Shen et al. [5] undertook a Chinese national investigation to map the geographical distribution of drinking water containing high levels of iodine. In the study, iodine concentrations above 300 *μ*g·L^−1^ were related to a high concentration of urinary iodine, resulting in a high prevalence of goiter (11.0%) in schoolchildren aged 8 to 10 years. Also, regions whose drinking water contained high and excess levels of iodine were identified. Based on the scenario of these specific regions, the study recommended three actions: (1) the sale of iodized salt should be stopped; (2) drinking water should be replaced and pipeline water with an adequate concentration of iodine should be supplied; and (3) iodine concentration in water should be monitored as well as the prevalence of goiter. Thus, the establishment of specific public policies on iodine in drinking water is driven by the identification of regions with insufficient, sufficient, or excess iodine in drinking water, as observed for China.

To date, no study has specifically monitored the concentration of iodine in Brazil's drinking water although some concentrations are well-known to be detrimental to nutritional status and thyroid function. However, most studies in the literature regarding the concentration of iodine in water are carried out in China [6–16].

Thus, the present study analyzed the concentration of iodine in the drinking water of all regions in Brazil and its possible contribution to iodine intake for different groups.

## 2. Materials and Methods

### 2.1. Analysis of Water Samples

Water samples from primary healthcare units (UBS) in seven municipalities and the Federal District, distributed across the five Brazilian macroregions (midwest, northeast, north, southeast, and south), were analyzed. The samples were collected during summer between 21/12/2018 and 19/03/2019. The locations, regions, and number of water samples are shown in Table 1.

The water samples were placed in 200 mL polyethylene bottles by local research team members and were kept frozen (−18°C) at the research centers of each location until dispatch to the Chemistry and Food Analysis laboratory of the Department of Food Technology, Federal University of Viçosa. All the samples (frozen) were transported in thermal boxes. Upon arrival at the laboratory, they were kept at 4°C until analysis.

### 2.2. Determination of Iodine Concentration in Drinking Water

The concentration of iodine in the water samples was quantified by a spectrophotometric method based on leuco crystal violet, which determines aqueous iodine in the form of elemental iodine and hypoiodous acid as described in the Standard Methods for the Examination of Water and Wastewater (4500-I B) [17].

An analytical curve was constructed daily for the analysis. For this purpose, a 10 mg iodine·L^−1^ solution was freshly prepared from a KI stock solution (1 mg iodine/mL).

Aliquots of 250 *μ*L to 2.500 *μ*L were pipetted from the 10 mg solution of iodine·L^−1^ and subsequently transferred to 100 mL volumetric flasks. The flasks were completed with ultrapure water. Then, 50 mL of the prepared solution was transferred to the other 100 mL volumetric flasks. Here, 1 mL of citric buffer solution and 0.5 mL of potassium peroxymonosulfate solution were added and stirred for approximately 1 minute. After that, 1 mL of leucocrystal violet was added and the volumetric flask was filled with ultrapure water. Thus, the standard iodine solutions for the analytical curve had concentrations of 0.0125; 0.0250; 0.0375; 0.0500; 0.0625; 0.0750; 0.0875; 0.1000; 0.1125; and 0.1250 mg of iodine·L^−1^.

The absorbance readings at 592 nm were done with an ultraviolet-visible spectrophotometer (model UV/VIS 9200, Rayleigh brand, 10 mm cuvette) at room temperature against a blank (concentration 0 mg·L^−1^) under the same conditions. From these readings, absorbance and iodine concentration data were plotted to construct an analytical curve with eleven concentrations, where each point was the average of two measurements. The construction of this curve validates the leucocrystal violet method for the determination of iodine concentration.

For the analysis of the water samples, 50 mL of the same was measured in a beaker and transferred to a 100 mL volumetric flask. In this flask, 1 mL of citric buffer solution and 0.5 mL of potassium peroxysulfate were added. The solution was stirred for approximately 1 minute. Then, 1 mL of leucocrystal violet was added and the volume was filled with ultrapure water. The analysis was conducted in triplicate, and the absorbance readings were taken within five minutes after the addition of leucocrystal violet for reliable results. The absorbance readings were performed under the same conditions as the standard solution. Based on the analytical curve, the results were expressed in*μ*g of iodine L^−1^.

With each new series of analyses, an iodine standard curve was built to guarantee the reliability of the results.

The legislation of China was adopted as a reference to assess the concentration of iodine in drinking water, with cutoff points being: <10 *μ*g/L (water with low iodine concentration); 10–150 *μ*g/L (water with adequate iodine concentration); >150 *μ*g/L (water with high iodine concentration), and >300 *μ*g/L (water with excess iodine) [18, 19].

### 2.3. Water Intake

Using the minimum and maximum iodine concentrations found in the drinking water from different locations, iodine intake in *μ*g was calculated for the following physiological groups: children, adolescents, adults/ elderly, pregnant women, and nursing mothers. For this purpose, the recommended water intake for each physiological group according to the United States Institute of Medicine was considered: 1 to 3 years (1.3 L); 4 to 8 years (1.7 L); 9 to 13 years (2.1 L); 14 to 18 years (2.3 L); 19 to over 70 years (2.7 L); pregnant women (3 L); and nursing mothers (3.8 L) [20]. The assessment considered iodine intake from drinking water among females in order to highlight pregnant women and nursing mothers, groups vulnerable to iodine deficiency.

To calculate the percentage of iodine present in the water samples from different locations and their possible contribution to iodine intake for different physiological groups, the estimated average requirement (EAR) of iodine by the Institute of Medicine was used as a reference [21].

### 2.4. Statistical Analysis

The concentration of iodine in the water samples was estimated by the average analytical curve adjusted by linear regression analysis (95% confidence) using the Statistical Analysis Systems (SAS) program (Statistical Analysis System-SAS Institute, Cary, NC, USA) version 9.2, licensed to the Federal University of Viçosa.

Data analysis was performed using the Statistical Package for Social Science (SPSS) version 21.0 and a significance level of 0.05. A descriptive statistical analysis of the data was conducted, and the results were expressed in absolute and relative frequencies. The concentration of iodine in drinking water was expressed as median, minimum, and maximum values.

### 2.5. Ethical Aspect

This study is part of a project entitled “Nutritional status of iodine, sodium, and potassium in the Brazilian maternal and infant group: a multicenter study,” which was approved by the Human Research Ethics Committee of the Federal University of Viçosa, approval number 2.496.986.

## 3. Results

**Figure 1:**
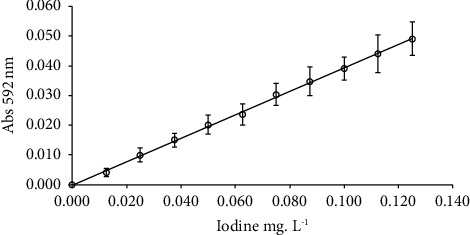
Adjusted analytical curve for iodine quantification in the water samples collected from locations in five Brazilian macroregions. Linear regression model ŷ = 0.3949 × x − 0.00015; *R*^2^ = 0.9989; *p* < 0.0001.

Figure 1 shows the analytical curve used for calculating iodine concentration in the water samples (mg·L^−1^).

Regarding the classification of iodine concentration in drinking water, 100% of the samples from the municipalities of Palmas (*n* = 10) and Vitória (*n* = 3) presented low iodine concentration. Eighty percent (80.0%) (*n* = 8) of the Pinhais samples had an adequate iodine level. It is important to note that none of the water samples from the different locations in the five Brazilian macroregions showed a high or excess iodine concentration following the classification of the Ministry of Health of China (Table 2).

The median, minimum, and maximum iodine concentrations in the water samples from different locations belonging to the Brazilian macroregions are shown in Table 3.

Significant differences were observed between the maximum and minimum iodine concentrations in water samples from the same location, being 44.32 *μ*g·L^−1^ in Pinhais; 27.86 *μ*g·L^−1^ in Viçosa; 12.66 *μ*g·L^−1^ in Rondonópolis; 11.82 *μ*g·L^−1^ in São Luís; 10.98 *μ*g·L^−1^ in Brasília; 10.14 *μ*g·L^−1^ in Macaé; 4.22 *μ*g·L^−1^ in Palmas; and 1.69 *μ*g·L^−1^ in Vitória.

Table 4 shows the percentages of iodine found in drinking water from different locations and their contribution (minimum and maximum) to micronutrient intake among different physiological groups.

The maximum concentrations of iodine found in the drinking water of Pinhais and Viçosa can contribute more than 50% of the recommended daily intake of iodine for all physiological groups (Table 4).

In the municipality of Viçosa, the drinking water can provide 73.9% of the recommended daily intake of iodine for individuals aged four to eight years, 81.2% for those aged nine to 13 years, and 80.3% for individuals aged 19 to 50 years. Regarding the municipality of Pinhais, the drinking water can contribute 93.0% of the recommended daily intake of iodine for all physiological groups, being more than 100.0% for individuals aged four to eight years (130.2%), nine to 13 years old (143.2%), 14 to 18 years old (120.5%), and 19 to 50 years old (141.5%) (Table 4).

## 4. Discussion

In Brazil, none of the water samples from the different locations of the five Brazilian macro regions showed a high (>150 *μ*g·L^−1^) or excess (>300 *μ*g·L^−1^) iodine concentration according to the classification of the Ministry of Health of China [19]. However, samples from identical locations presented significant differences in minimum and maximum concentrations, resulting in discrepant contributions to iodine intake among the different physiological groups.

The municipalities of Pinhais and Viçosa had the highest percentage of iodine in drinking water and, consequently, the highest contributions to iodine intake. The maximum concentration of iodine in the drinking water of these places could contribute more than 50% of the recommended intake of iodine for all physiological groups. Besides iodine ingested from drinking water, it is important to note the iodine ingestion from iodized salt and food. Considering a scenario where an eight-year-old child from the municipality of Pinhais drinks 1.7 L of water per day, he would have an iodine intake of 84.6 *μ*g. Given a recommended daily salt intake of 5 grams per day (Brazilian Ministry of Health) [22], with an average iodine concentration of 30 mg/kg of salt, one can anticipate an iodine intake of 150.0 *μ*g. Therefore, the child's total iodine intake is 234.6 *μ*g per day, which would be 3.6 times higher than the estimated average requirement (65 *μ*g/day) of iodine for children aged one to eight years [21].

However, if we consider salt consumption estimated by the Family Budget Survey (POF) [23], of 12 grams of salt per day, then the child's iodine intake would be 360.0 *μ*g/day. Thus, maintaining 1.7 L of drinking water, the total consumption of iodine is now 444.6 *μ*g/day. This points to excessive intake (≥300 *μ*g/day) of iodine, which is almost seven times (6.8) higher than the estimated average requirement of iodine for this physiological group.

Data from the National Survey for the Evaluation of the Impact of Salt Iodization (PNAISAL) conducted with 19,600 schoolchildren between six and 14 years old showed that 25.2% of them had urinary iodine concentrations between 200 and 299 *μ*g/day (more than the recommended intake) and 44.6% had urinary iodine concentrations ≥300 *μ*g/day (excessive intake) [24]. Although we did not evaluate urinary iodine, the example of the eight-year-old child suggests that iodine intake can be greater than the recommended value and excessive if water with a higher concentration of iodine is ingested in addition to the use of iodized salt (5 grams or 12 grams of salt per day). This excessive iodine intake can cause goiter, hypothyroidism and overt hyperthyroidism, hypothyroidism, subclinical hyperthyroidism, autoimmune thyroid disease, iodine allergies, and decreased intelligence [1, 25–28].

Regarding an adult residing in Pinhais or Viçosa who consumes 2.7 mL of water, the daily iodine intake will be 134.4 *μ*g or 76.3 *μ*g, respectively. An intake of 5 grams of salt per day, according to the recommendation of the Ministry of Health [22], contributes to a daily iodine intake of 150 *μ*g. Accordingly, the total iodine intake is 234.4 *μ*g/day and 226.3 *μ*g/day for Pinhais and Viçosa, respectively. This daily intake of iodine considering the consumption of drinking water with a higher concentration of iodine plus iodized salt intake would represent a more than adequate intake of 200–299 *μ*g/day. On the other hand, considering 12 grams of salt per day is normally consumed by Brazilians [23], the individual would have an iodine intake of 360.0 *μ*g/day, totaling 494.4 *μ*g/day (Pinhais) and 436.3 *μ*g/day (Viçosa), which represents excessive intake of iodine (≥300 *μ*g/day) being 5.2 and 4.6 times higher than the estimated average requirement of this group (95 *μ*g/day) [21] exposing the adult population to the risks associated with excess iodine intake.

Pregnant women and nursing mothers rarely have excessive iodine intake because their physiological conditions demand greater iodine levels [29]. In this study, pregnant women and nursing mothers in the municipality of Viçosa would have a daily iodine intake of 84.7 *μ*g and 107.3 *μ*g, respectively, if they drank 3 L (pregnant women) and 3.8 L (nursing mothers) of water containing a higher concentration of iodine. If we consider water intake and the ingestion of iodized salt (5 grams of salt per day), pregnant women and nursing mothers would have a daily iodine intake of 234.7 *μ*g and 257.3 *μ*g, respectively, exceeding the estimated average requirement of iodine, being 160 *μ*g/day for pregnant women and 200 *μ*g/day for nursing mothers [21]. In the case of 12 grams of salt per day and water intake, pregnant women would have a daily iodine intake of 444.7 *μ*g and nursing mothers 467.3 *μ*g, being almost 2.8 and 2.4 times higher, respectively, than the estimated average required for these population groups.

In the municipality of Pinhais, pregnant women and nursing mothers would have an iodine intake of 299.3 *μ*g/day and 339.1 *μ*g/day, considering the consumption of drinking water with a higher concentration of iodine and the ingestion of 5 grams of salt daily, exceeding the estimated average requirement for these groups [22].

On the other hand, if we consider the ingestion of 12 grams of salt per day plus the consumption of drinking water, pregnant women and nursing mothers in Pinhais would have a daily iodine intake of 509.3 *μ*g and 549.1 *μ*g, respectively, representing an excessive intake, being almost 3.2 and 2.8 times higher than the estimated average iodine requirement, respectively.

An important aspect to be considered in the interpretation of our results refers to the choice of the EAR for calculating the percentage of iodine present in the water samples from different locations and their possible contribution to iodine intake for different physiological groups. The EAR choice is to be considered as a conservative and hypothetical requirement in our study, considering that there is variability in the individual requirements, which is unknown [30].However, this should give us an idea of the contribution of the iodine content in the water in relation to these hypothetical needs. Another possibility would be to use the Recommended Dietary Allowance Intake (RDA) for this evaluation. In such a case, the percentage of iodine present in the water would therefore contribute less to the individual requirements.

Given the above considerations, the concentration of iodine in drinking water must be taken into account when assessing the nutritional status of iodine among the population. Depending on the location, excessive iodine intake can occur in the different physiological groups which can change the health status of the population.

## 5. Conclusions

The concentrations of iodine in the water samples showed discrepant contributions to iodine intake, and in some places they could contribute to excess intake among the different physiological groups.

Therefore, monitoring the concentration of iodine in drinking water from different locations is recommended for the establishment of specific strategies according to the location and physiological group. This approach may prevent excessive iodine intake and consequent changes in the functioning of the thyroid gland that may impact population health.

## Figures and Tables

**Table 1:** Location, Brazilian region, and number of water samples analyzed at each site.

Location	Brazilian region	Number of samples
Brasília	Midwest	12
Rondonópolis	Midwest	15
São Luís	Northeast	12
Palmas	North	10
Macaé	Southeast	9
Viçosa	Southeast	14
Vitória	Southeast	3
Pinhais	South	10

**Table 2:** Percentage of iodine based on the classification of iodine concentration in the water samples collected from Primary Healthcare Units (PHU) in locations situated in the macro regions of Brazil.

Location/region	Iodine concentration in water							
	Low		Adequate		High		Excess	
	<10 *μ*g·L^−1^^*∗*^		10–150 *μ*g·L^−1^^*∗*^		>150 *μ*g·L^−1^^*∗∗*^		>300 *μ*g·L^−1^^*∗∗*^	
	N	%	n	%	N	%	n	%
Brasília (midwest)	10	83.3	2	16.7	—	—	—	—
Rondonópolis (midwest)	14	93.3	1	6.7	—	—	—	—
São Luís (northeast)	11	91.7	1	8.3	—	—	—	—
Palmas (north)	10	100.0	—	—	—	—	—	—
Macaé (Southeast)	7	77.8	2	22.2	—	—	—	—
Viçosa (southeast)	12	85.7	2	14.3	—	—	—	—
Vitória (Southeast)	3	100.0	—	—	—	—	—	—
Pinhais (south)	2	20.0	8	80.0	—	—	—	—

^*∗*^[18]; ^*∗∗*^[19].

**Table 3:** Median, minimum, and maximum iodine concentrations in water samples from Primary Healthcare Units (PHUs) in macroregions of Brazil.

Drinking water	Location/region							
	Midwest		Northeast	North	Southeast			South
	Brasília	Rondonópolis	São Luís	Palmas	Macaé	Viçosa	Vitória	Pinhais
Median concentration (*μ*g·L^−1^)	1.65	3.76	1.65	2.49	2.91	3.33	2.07	17.26
Minimum concentration (*μ*g·L^−1^)	0.38	0.38	0.38	0.38	1.22	0.38	1.22	5.45
Maximum concentration (*μ*g·L^−1^)	11.36	13.04	12.20	4.60	11.36	28.24	2.91	49.77

**Table 4:** Values of minimum and maximum iodine concentrations (*μ*g), percentages of iodine in drinking water from different locations, and their minimum and maximum contribution to iodine intake among different physiological groups.

Physiological groups	Estimated average requirement (EAR)^*∗*^	Values of minimum and maximum (*μ*g), percentages of iodine in drinking water, and their minimum and maximum contribution to iodine intake							
		Location							
		Brasília		Rondonópolis		São Luís		Palmas	
		Minimum^1^	Maximum^2^	Minimum	Maximum	Minimum	Maximum	Minimum	Maximum
1–3 years	65 *μ*g	0.49 *μ*g 0.75%	14.77 *μ*g 22.72%	0.49 *μ*g 0.75%	16.95 *μ*g 26.08%	0.49 *μ*g 0.75%	15.86 *μ*g 24.40%	0.49 *μ*g 0.75%	5.98 *μ*g 9.20%
4–8 years	65 *μ*g	0.65 *μ*g 1.00%	19.31 *μ*g 29.71%	0.65 *μ*g 1.00%	22.17 *μ*g 34.11%	0.65 *μ*g 1.00%	20.74 *μ*g 31.91%	0.65 *μ*g 1.00%	7.82 *μ*g 12.03%
9–13 years	73 *μ*g	0.80 *μ*g 1.09%	23.86 *μ*g 32.68%	0.80 *μ*g 1.09%	27.38 *μ*g 37.50%	0.80 *μ*g 1.09%	25.62 *μ*g 35.09%	0.80 *μ*g 1.09%	9.68 *μ*g 13.26%
14–18 years	95 *μ*g	0.87 *μ*g 0.92%	26.13 *μ*g 27.51%	0.87 *μ*g 0.92%	29.99 *μ*g 31.57%	0.87 *μ*g 0.92%	28.06 *μ*g 29.53%	0.87 *μ*g 0.92%	10.58 *μ*g 11.13%
19–50 years, 51–70 years, and >70 years	95 *μ*g	1.03 *μ*g 1.08%	30.67 *μ*g 32.28%	1.03 *μ*g 1.08%	35.21 *μ*g 37.06%	1.03 *μ*g 1.08%	32.94 *μ*g 34.67%	1.03 *μ*g 1.08%	12.42 *μ*g 13.07%
Pregnant women (14–50 years)	160 *μ*g	1.14 *μ*g 0.71%	34.08 *μ*g 21.30%	1.14 *μ*g 0.71%	39.12 *μ*g 24.45%	1.14 *μ*g 0.71%	36.60 *μ*g 22.88%	1.14 *μ*g 0.71%	13.80 *μ*g 8.63%
Nursing mothers (14–50 years)	200 *μ*g	1.44 *μ*g 0.72%	43.17 *μ*g 21.59%	1.44 *μ*g 0.72%	49.55 *μ*g 24.78%	1.44 *μ*g 0.72%	36.60 *μ*g 18.30%	1.44 *μ*g 0.72%	17.48 *μ*g 8.74%

Physiological groups	Estimated average requirement (EAR)^*∗*^	Values of minimum and maximum (μg), percentages of iodine in drinking water and their minimum and maximum contribution to iodine intake							
		Location							
		Macaé		Viçosa		Vitória		Pinhais	
		Minimum^*1*^	Maximum^*2*^	Minimum	Maximum	Minimum	Maximum	Minimum	Maximum
1–3 years	65 *μ*g	1.59 *μ*g 2.45%	14.77 *μ*g 22.72%	0.49 *μ*g 0.75%	36.71 *μ*g 56.47%	1.59 *μ*g 2.45%	3.78 *μ*g 5.82%	7.09 *μ*g 10.91%	64.70 *μ*g 99.54%
4–8 years	65 *μ*g	2.07 *μ*g 3.18%	19.31 *μ*g 29.71%	0.65 *μ*g 1.00%	48.00 *μ*g 73.85%	2.07 *μ*g 3.18%	4.95 *μ*g 7.61%	9.27 *μ*g 14.26%	84.61 *μ*g 130.17%
9–13 years	73 *μ*g	2.56 *μ*g 3.51%	23.86 *μ*g 32.68%	0.80 *μ*g 1.09%	59.30 *μ*g 81.23%	2.56 *μ*g 3.51%	6.11 *μ*g 8.37%	11.45 *μ*g 15.68%	104.52 *μ*g 143.18%
14–18 years	95 *μ*g	2.81 *μ*g 2.96%	26.13 *μ*g 27.51%	0.87 *μ*g 0.92%	64.95 *μ*g 68.37%	2.81 *μ*g 2.96%	6.69 *μ*g 7.04%	12.54 *μ*g 13.20%	114.47 *μ*g 120.49%
19–50 years, 51–70 years, and >70 years	95 *μ*g	3.29 *μ*g 3.46%	30.67 *μ*g 32.28%	1.03 *μ*g 1.08%	76.25 *μ*g 80.26%	3.29 *μ*g 3.46%	7.86 *μ*g 8.27%	14.71 *μ*g 15.48%	134.38 *μ*g 141.45%
Pregnant women (14–50 years)	160 *μ*g	3.66 *μ*g 2.29%	34.08 *μ*g 21.30%	1.14 *μ*g 0.71%	84.72 *μ*g 52.95%	3.66 *μ*g 2.29%	8.73 *μ*g 5.46%	16.35 *μ*g 10.22%	149.31 *μ*g 93.32%
Nursing mothers (14–50 years)	200 *μ*g	4.64 *μ*g 2.32%	43.17 *μ*g 21.59%	1.44 *μ*g 0.72%	107.31 *μ*g 53.66%	4.64 *μ*g 2.32%	11.06 *μ*g 5.53%	20.71 *μ*g 10.36%	189.13 *μ*g 94.57%

^*∗*^ [21]. Values for individuals aged 0–12 months were not determined. ^1^Minimum: minimum concentration of iodine in drinking water based on location; ^2^Maximum: maximum concentration of iodine in drinking water based on location.

## Data Availability

Data are available in Excel spreadsheets.

## Supplementary Materials

Supplementary Materials Two files have been included in “Supplementary Files”. The files refer to the databases of the analysis of iodine in water in the summer season (File 1) and data from the analytical curves to build the adjusted analytical curve (File 2).

## References

[1] Teng W., Shan Z., Teng X., Guan H., Li Y. (2006). Effect of iodine intake on thyroid diseases in China. *New England Journal of Medicine*.

[2] World Health Organization (2007). *Assessment of Iodine Deficiency Disorders and Monitoring Their Elimination: A Guide for Programme Managers: a Guide for Programme Managers*.

[3] BRASIL Ministério da Saúde (2007). *Cadernos de Atenção Básica: Carência de Micronutrientes. Brasília*.

[4] Zhao H., Tian Y., Liu Z., Li X., Feng M., Huang T. (2014). Correlation between iodine intake and thyroid disorders: a cross-sectional study from the South of China. *Biological Trace Element Research*.

[5] Shen H., Liu S., Sun D. (2011). Geographical distribution of drinking-water with high iodine level and association between high iodine level in drinking-water and goitre: a Chinese national investigation. *British Journal of Nutrition*.

[6] Li M., Liu D. R., Qu C. Y. (1987). Endemic goitre in central China caused by excessive iodine intake. *Lancet*.

[7] Zhao J., Chen Z., Maberly G. (1998). Iodine-rich drinking water of natural origin in China. *The Lancet*.

[8] Zhao J., Wang P., Shang L., Sullivan K. M., van der Haar F., Maberly G. (2000). Endemic goiter associated with high iodine intake. *American Journal of Public Health*.

[9] Andersen S., Guan H., Teng W., Laurberg P. (2009). Speciation of iodine in high iodine groundwater in China associated with goitre and hypothyroidism. *Biological Trace Element Research*.

[10] Lv S., Zhao J., Xu D. (2012). An epidemiological survey of children’s iodine nutrition and goitre status in regions with mildly excessive iodine in drinking water in Hebei Province, China. *Public Health Nutrition*.

[11] Tang Q., Xu Q., Zhang F. (2013). Geochemistry of iodine-rich groundwater in the taiyuan basin of central shanxi province, North China. *Journal of Geochemical Exploration*.

[12] Zhang E., Wang Y., Qian Y. (2013). Iodine in groundwater of the North China Plain: spatial patterns and hydrogeochemical processes of enrichment. *Journal of Geochemical Exploration*.

[13] Li J., Wang Y., Xie X., DePAOLO D. J. (2016). Effects of water-sediment interaction and irrigation practices on iodine enrichment in shallow groundwater. *Journal of Hydrology*.

[14] Cui S. L., Liu P., Su X. H., Liu S. J. (2017). Surveys in areas of high risk of iodine deficiency and iodine excess in China, 2012-2014: current status and examination of the relationship between urinary iodine concentration and goiter prevalence in children aged 8-10 years. *Biomedical and Environmental Sciences: Biomedical and Environmental Sciences*.

[15] Xue X., Li J., Xie X. (2019). Effects of depositional environment and organic matter degradation on the enrichment and mobilization of iodine in the groundwater of the North China Plain. *Science of the Total Environment*.

[16] Yang Z., Wang C., Nie Y. (2021). Investigation on spatial variability and influencing factors of drinking water iodine in Xinjiang, China. *PLoS One*.

[17] (2020). Standard methods for the examination of water and wastewater. https://www.mwa.co.th/download/file_upload/SMWW_4000-6000.pdf.

[18] Ministry of Health of China (2003). Determination and classification of the areas of high water iodine and the endemic areas of iodine excess goiter. *National Standard of the People’s Republic of China*.

[19] Ministry of Health of China (2009). *Delimitation for the Endemic Areas of Iodine Deficiency Disorders*.

[20] Institute of Medicine–IOM (2004). *Dietary Reference Intakes for Water, Potassium, Sodium Chloride, and Sulfate*.

[21] Institute of Medicine–IOM (2001). *Dietary Reference Intakes for Vitamin A, Vitamin K, Arsenic, Boron, Copper, Iodine, Manganese, Molybdenum, Nickel, Silicon, Vanadium and Zinc*.

[22] BRASIL Ministério da Saúde (2006). *Secretaria de Atenção à Saúde. Coordenação - Geral da Política de Alimentação e Nutrição. Guia alimentar para a população brasileira: promovendo a alimentação saudável*.

[23] Instituto Brasileiro DE Geografia E Estatística (2011). *Pesquisa de Orçamentos Familiares 2008-2009: Análise do consumo alimentar pessoal no Brasil*.

[24] Santos I. S., Cesar J. A. (2016). *Pesquisa Nacional para Avaliação do Impacto da Iodação do Sal (PNAISAL)*.

[25] Yu Z., Zhu H., Chen Z. (1999). Progress in endemic iodine excess goiter. *China Journal Epidemiology*.

[26] Bournaud C., Orgiazzi J. J. (2003). Iodine excess and thyroid autoimmunity. *Journal of Endocrinological Investigation*.

[27] Li Y., Teng D., Shan Z. (2008). Antithyroperoxidase and antithyroglobulin antibodies in a five-year follow-up survey of populations with different iodine intakes. *Journal of Clinical Endocrinology and Metabolism*.

[28] Liu H. L., Lam L. T., Zeng Q., Han S. q., Fu G., Hou C. c. (2008). Effects of drinking water with high iodine concentration on the intelligence of children in Tianjin, China. *Journal of Public Health*.

[29] Saraiva D. A., Morais N. A. d. O. e. S. d., Martins Corcino C. (2018). Iodine status of pregnant women from a coastal Brazilian state after the reduction in recommended iodine concentration in table salt according to governmental requirements. *Nutrition*.

[30] Institute of Medicine–IOM (2000). Subcommittee on interpretation and uses of dietary reference intakes, institute of medicine (US) standing committee on the scientific evaluation of dietary reference intakes. *DRI Dietary Reference Intakes: Applications In Dietary Assessment*.

